# Impact of sports participation on genu varum among youth: a systematic review

**DOI:** 10.1093/bmb/ldag033

**Published:** 2026-10-05

**Authors:** Ali Işın, Eren Akdağ, Burhan Demirkıran, Dennis Caine

**Affiliations:** Department of Coaching Education, Faculty of Sport Sciences, Akdeniz University, Dumlupınar Bulvarı, Konyaaltı, Antalya 07070, Türkiye; School of Population Health, Faculty of Medicine and Health, UNSW Sydney, 55 Botany Street, Randwick, NSW 2031, Australia; Department of Coaching Education, Faculty of Sport Sciences, Akdeniz University, Dumlupınar Bulvarı, Konyaaltı, Antalya 07070, Türkiye; Institute of Health Sciences, Akdeniz University, Dumlupınar Bulvarı, Konyaaltı, Antalya 07070, Türkiye; Department of Education, Health and Behavior Studies, University of North Dakota, 231 Centennial Dr Stop 7189, Grand Forks, ND 58202-7189, United States

**Keywords:** genu varum, bowlegs, lower limb malalignment, varus deformity, adolescent, athlete

## Abstract

**Introduction:**

Genu varum occurs more frequently among young male athletes involved in high levels of physical activity, particularly adolescent male soccer players when compared to the general population. This study provides a contemporary examination of the relationship between genu varum and participation in youth sport.

**Sources of data:**

A systematic review was performed following Preferred Reporting Items for Systematic Reviews and Meta-Analysis guidelines. The literature search was conducted in Web of Science, Scopus, PubMed, Embase, and CINAHL databases.

**Areas of agreement:**

Genu varum is more prevalent among athletes than among age-matched non-athlete controls and has been reported more often in male soccer players, with overloading of the proximal medial tibial growth plate proposed as a possible contributing mechanism.

**Areas of controversy:**

Intercondylar-intermalleolar distance differed in younger age groups with some reporting negative values and some reporting positive values.

**Growing points:**

In recent years, there has been a marked improvement in the methods used to assess lower limb malalignment in young athletes. Clinical measures are simple but lack detail; whole-leg radiographs are increasingly used to capture these details.

**Areas timely for developing research:**

The lack of longitudinal data examining changes in lower extremity alignment with sports loading during the growth period is an important deficiency of the current research. Long-term follow-up studies are needed to clarify the nature and incidence of genu varum in young athletes, including females. Furthermore, the relationship between genu varum and non-soccer sports should be investigated so the movement and loading characteristics specific to different sports need to be evaluated.

## Introduction

Genu varum, commonly known as “bowlegs,” is a lower extremity malalignment characterized by the knees bending outward while the ankles are close together [1]. This deformity is particularly seen in infants and young children during development. Children may suffer from knee varus that decreases with age from birth, and this deformity tends to resolve by approximately 2 years of age [2]. Various conditions, such as Blount’s disease, rickets, and metabolic deficiencies, can contribute to the development of genu varum outside the normal developmental process [3]. A growing body of research shows that genu varum occurs more frequently among young male athletes involved in high levels of physical activity, particularly adolescent soccer players when compared to the general population [4–8].

Individuals who participate in sports throughout childhood, adolescence, and adulthood engage in more physical activity, report better health and well-being, have a healthier body composition, and report less mental ill-being over time compared to nonparticipants [9]. However, participation in sports at an early age may also cause changes in posture alignment [10] and skeletal growth [11] in youth whose bone and muscle systems are still developing. These posture changes and overuse injury of the epiphyseal primary physis may also contribute to the development of deformities such as genu varum [8, 11]. This is concerning as genu varum predisposes athletes to abnormal biomechanical loading of the medial tibiofemoral joint, traumatic chondral and meniscal lesions, and premature medial joint osteoarthritis [4, 12].

The physes near the knee joint play a crucial role in leg length and mechanical axis, making them susceptible to athletic overload [13]. A recent systematic review of stress injury involving the primary growth plates of epiphyses reveals growing evidence of physeal stress injury (PSI) and infrequent growth disturbance involving the epiphyseal growth plate complex of the distal femur and proximal tibia in youth athletes representing a variety of sports, including soccer [11]. Although incidence data related to these injuries are lacking, cross-sectional studies indicate a relatively high prevalence of genu varum in young athletes, particularly soccer players [6, 7, 14]. It is postulated that the high frequency of genu varum in youth athletes is the result of repetitive rotation and compression forces affecting the growth plates of knee joints [12]. However, there may be other, possibly pre-existent factors, where those with borderline or genu varum alignment are preferentially selected, because it offers improved balance and athletic performance [5, 15].

Against this background, we believe that a systematic review describing the present status of knowledge on the nature, frequency and etiology of genu varum in youth sports is both timely and useful. This systematic review aims to examine the relationship between genu varum and participation in youth sports; compare the measurement methods used to assess genu varum; determine the prevalence of genu varum by sport type; and identify gaps in the existing literature and provide recommendations for future research.

## Materials and methods

The protocol for this systematic review was prospectively registered with PROSPERO (CRD420251232026) and conducted according to the Preferred Reporting Items for Systematic Reviews and Meta-Analysis (PRISMA) guidelines [16].

### Literature search

The literature search was conducted in the Web of Science, Scopus, PubMed, Embase, and CINAHL databases from inception to 24 November 2025.

Specific search strategies for each database were created using MeSH terms and keywords, and Boolean operators. The terms “genu varum,” “bowlegs,” “varus knee malalignment,” “lower leg alignment,” “lower leg malalignment,” “leg deformity,” “lower limb deformity,” “lower limb malalignment,” “youth,” “young,” “adolescent,” “sport,” “soccer,” “football,” “basketball,” “volleyball,” “athlete” were among the main keywords used in the search. Detailed search strategies in each database are available in Supplementary Table S1.

### Study selection

The screening process was conducted by dual independent authors (A.I. and B.D.). Articles that passed the title and abstract screening were included in the full text screening. Any conflicts were resolved by the lead author (A.I.). Study screening was carried out using Covidence systematic review software [17]. The inclusion and exclusion criteria are presented in Table 1.

**Table 1:** Inclusion and exclusion criteria.

Inclusion criteria	Exclusion criteria
Peer-review literature Original research Athletes in the youth and adolescent age group (aged <18 years) Athletes who are male, female, or both Athletes who regularly participate in any sport Clinically or radiologically measured genu varum or leg axis deformities Written in English	Non-peer-reviewed Non-original research (i.e. literature reviews, editorial articles, proceeding paper, meeting abstract, letter, and commentary articles) Studies in which genu varum or leg axis deformities were not measured Studies not defined as a sport discipline

### Data extraction

The data extraction form was created on Microsoft Excel and the data extraction process for all studies was carried out performed independently by two authors (E.A. and B.D.) using this standard form. The following information was included: author’s name, year of publication, study design, country, participants (sample size, sex, age) type of sport, measurement method used (clinical or radiologic), measured parameters, and degree of genu varum. Differences between the authors were reviewed by the lead author (A.I.) and resolved by consensus. During the data extraction process, authors of relevant studies were contacted when necessary to request additional information or to address missing data.

### Quality appraisal

The methodological quality of included studies was assessed using the Joanna Briggs Institute (JBI) Critical Appraisal Tools, which provide study design-specific checklists for risk of bias assessment. Each study was assessed by two independent authors (E.A. and B.D.) using the relevant JBI checklist (8-item checklist for cross-sectional studies, 10-item checklist for case control and case-series studies and 11-item checklist for cohort studies). Any disagreements were resolved through discussion or consultation with a lead author (A.I.). JBI methodological quality assessment of the included studies is available in Supplementary Table S2.

## Results

### Study selection

A total of 1519 studies were identified through Web of Science, Scopus, PubMed, Embase, and CINAHL database searches. After excluding 598 duplicate studies, title-abstract screening was performed on 921 studies, and then 60 studies were included in the full-text review. We excluded 44 studies that did not meet the inclusion criteria, and 16 studies were included in this systematic review (Fig. 1). Among the 16 included studies, publication dates ranged from 2006 to 2025.

**Figure 1:**
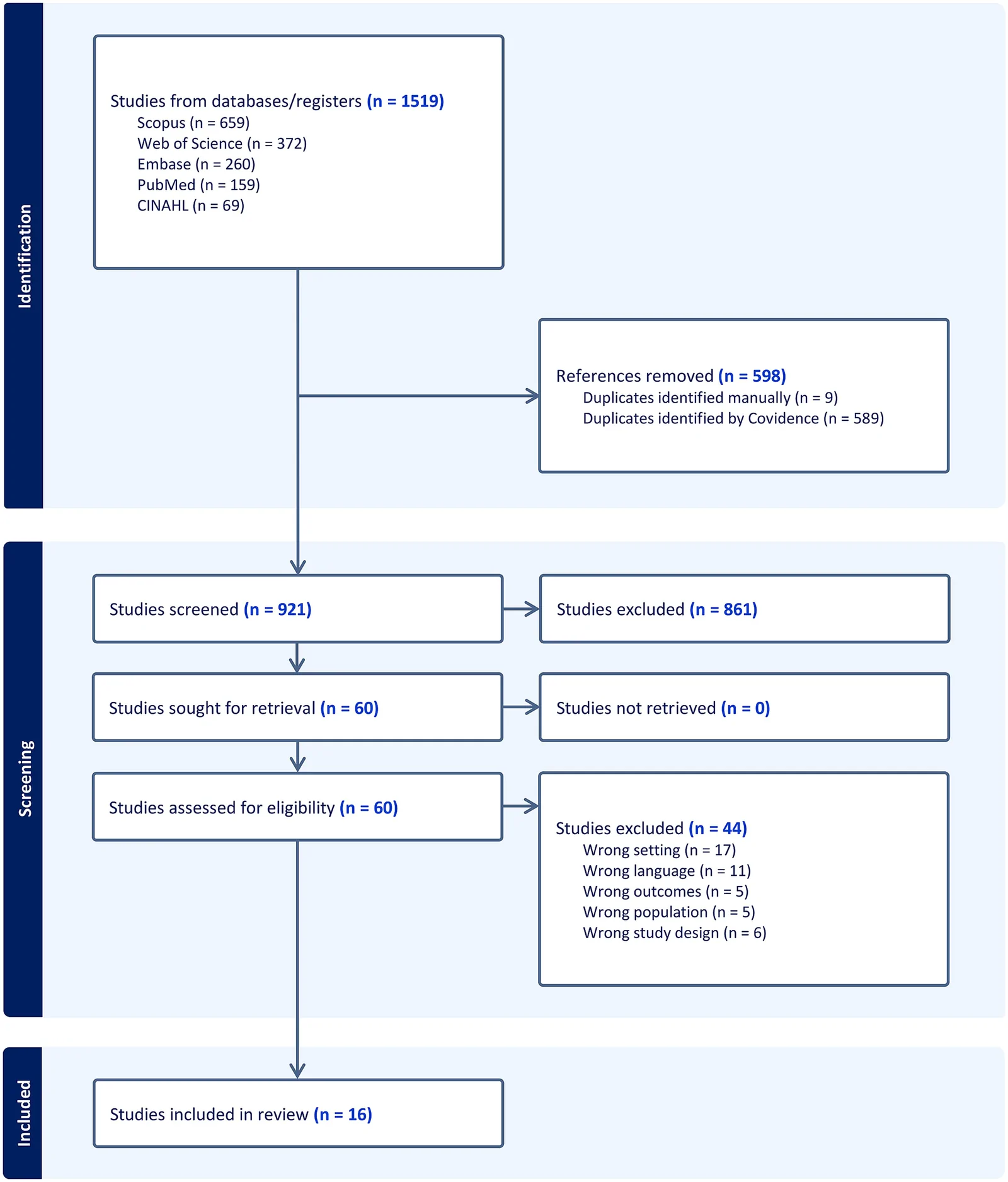
PRISMA flow diagram.

### Study designs

Characteristics of studies included in the systematic review are summarized in Table 2. There are 12 cross-sectional studies [5–7, 15,18–25], 1 diagnostic study (Level of Evidence III) [4], 1 prospective case–control study (Level of Evidence III) [26], a 4-month single-center case series [27], and 1 retrospective cohort study [14]. The mean JBI methodological quality assessment score of the included studies is 88.82%.

**Table 2:** Characteristics of the studies included in the systematic review.

Author (year)	Study population	Sample size (*n*)	Age (year)	Sex	Measurement method		Measured parameters
Asadi *et al*. (2015) [7]	Soccer players and non-soccer players	1500	10–18	M	Clinical	-	ICD-IMD
Colyn *et al*. (2016) [4]	Soccer players, other athletes, and non-athletic controls	200	10–14	M/F	Radiological	A full-leg radiographs	HKA, MPTA, mLDFA, LPFA, MNSA, KVPA, Bowing
De Cock *et al*. (2018) [18]	Weight-bearing sports	1008	10–17	M/F	Clinical	Calliper	ICD-IMD
Rezende *et al*. (2011) [19]	Soccer players and non-soccer players	128	14–17	M	Clinical	Goniometer	ICD-IMD
Fernquest *et al*. (2023) [5]	Soccer players, male controls, and female controls	125	11–21^a^	M/F	Radiological	A full leg length MRI	HKA, MPTPhyA, LDFPhyA
Işın *et al*. (2025) [26]	Soccer players with genu varum and without genu varum	36	10–19	M	Clinical and Radiological	Calliper and a full leg length MRI	ICD-IMD, MAD, mLPFA, mLDFA, MPTA, LDTA, HKA, Q angle, TT, FA, TT-TG, TT-PCL
Işın and Melekoğlu (2020) [6]	Soccer players and non-athletes	474	10–18	M	Clinical	Calliper and standardized digital frontal-plane photograph	ICD-IMD, Q angle
Krajnc *et al*. (2015) [27]	Soccer players	11	14	M	Clinical	Calliper	ICD-IMD
Memmel *et al*. (2023) [20]	Soccer players and referees	57	15–19	M	Clinical	Calliper and standardized digital frontal-plane photograph	ICD-IMD, HKA
Memmel *et al*. (2024) [21]	Soccer players	207	7–18	M	Clinical	Calliper	ICD-IMD
Schulz *et al*. (2025) [22]	Soccer players	381	10–23^a^	M	Clinical	Calliper	ICD-IMD
Steffen *et al*. (2025) [23]	Soccer players	168	10–21^a^	M	Clinical	Tape measure and camera-based posture and gait analysis system	ICD-IMD, MAA
Thijs *et al*. (2012) [15]	Athletes and non-athletes	521	7–18	M	Clinical	Calliper	ICD-IMD
Witvrouw *et al*. (2009) [14]	Soccer player and non-soccer players	794	8–18	M	Clinical	Calliper	ICD-IMD
Yaniv *et al*. (2006) [24]	Soccer players and tennis players	174	10–21^a^	M	Clinical	Flexible ban	ICD-IMD
Žlak *et al*. (2024) [25]	Soccer player and ice hockey players	128	12–16	M	Clinical	Calliper	ICD-IMD

^a^Only data under the age of 18 are included.

ICD-IMD, intercondylar-intermalleolar distance; HKA, hip–knee–ankle angle; MPTA, medial proximal tibial angle; mLDFA, mechanical lateral distal femoral angle; LPFA, lateral proximal femoral angle; MNSA, medial neck-shaft angle; KVPA, The knee valgus proximal angle; MPTPhyA, medial proximal tibial physeal angle; LDFPhyA, lateral distal femoral physeal angle; MAD, mechanical axis deviation; mLPFA, mechanical lateral proximal femoral angle; LDTA, lateral distal tibial angle; Q angle, Quadriceps femoris angle; TT, tibial torsion; FA, femoral anteversion; TT-TG, tibial tubercle-trochlear groove distance; TT-PCL, tibial tubercle-posterior cruciate ligament; MAA, The mechanical femorotibial axis angle

### Participation and measurement method

The 16 studies included in the systematic review contain a total of 5912 participants. Of these, 13 studies enrolled exclusively male athletes [6, 7, 14, 15,19–27], whereas the remaining 3 evaluated both male and female participants [4, 5, 18]. Methodologically, 13 studies used only clinical measurement methods [6, 7, 14, 15,18–25, 27], 2 used only radiologic methods [4, 5], and 1 used both [26]. Regarding knee alignment assessment, intercondylar distance-intermalleolar distance (ICD-IMD) was measured in 14 of the 16 studies. Notably, most of these studies (*n* = 10) reported using a caliper for assessment, which is a standard clinical method. Furthermore, a substantial proportion of the included studies (*n* = 14) focused on soccer players as the main study population (Table 2). Thus, the available evidence was predominantly derived from soccer-specific populations, with limited representation from other youth sports.

### Intercondylar-intermalleolar distance outcomes

Table 3 shows the ICD-IMD measures reported in the included studies. In studies using a control group, soccer players had higher ICD-IMD values than non-soccer players [6, 7, 14, 24]. In soccer players aged 15–18 years, these values increased significantly. In cross-sectional studies without a control group, a trend of increasing varus axis deviation with age was observed [21–23, 27]. In general, studies indicate a more pronounced genu varum tendency in soccer players.

**Table 3:** Intercondylar-intermalleolar distance outcomes reported in the included studies.

Author (year)	Group	ICD-IMD (mean ± SD)	Control	ICD-IMD (mean ± SD)	Main outcomes
Asadi *et al*. (2015) [7]	SP	3.01 ± 1.2	NSP	2.28 ± 1.08	Compared to NSP, SP has a higher ICD-IMD.
Işın and Melekoğlu (2020) [6]	SP	2.02 ± 1.80	NSP	0.29 ± 0.60	SP was found to be higher than NSP in all age groups. In SP, the highest ICD-IMD was found in the 16-year-old age group. ICD-IMD increased with age.
	10 yr SP	0.40 ± 0.48	10 yr NSP	0.02 ± 0.05	
	11 yr SP	0.55 ± 0.56	11 yr NSP	0.15 ± 0.33	
	12 yr SP	0.46 ± 0.43	12 yr NSP	0.07 ± 0.15	
	13 yr SP	1.02 ± 1.12	13 yr NSP	0.41 ± 0.65	
	14 yr SP	1.92 ± 1.30	14 yr NSP	0.14 ± 0.35	
	15 yr SP	2.21 ± 1.80	15 yr NSP	0.21 ± 0.35	
	16 yr SP	3.54 ± 1.71	16 yr NSP	0.49 ± 0.83	
	17 yr SP	2.98 ± 1.55	17 yr NSP	0.39 ± 0.61	
	18 yr SP	3.22 ± 2.16	18 yr NSP	0.41 ± 0.58	
Işın *et al*. (2025) [26]	SP with genu varum	5.65 ± 1.00	SP without genu varum	1.87 ± 1.20	ICD-IMD were found to be higher in SP with genu varum.
Memmel *et al*. (2023) [20]	SP	1.70 ± 2.50	Referees	1.30 ± 2.70	There is no difference between SP and referees in terms of ICD-IMD.
Thijs *et al*. (2012) [15]	7–9 yr sporting	−1.32 ± 0.34	7–9 yr non-sporting	−2.15 ± 0.55	ICD-IMD of sporting boys in the 13–15 and 16–18 age groups were higher than the control group in their age groups.
	10–12 yr sporting	−1.91 ± 0.44	10–12 yr non-sporting	−3.26 ± 0.62	
	13–15 yr sporting	0.49 ± 0.29	13–15 yr non-sporting	−0.63 ± 0.32	
	16–18 yr sporting	2.47 ± 0.25	16–18 yr non-sporting	0.50 ± 0.32	
Witvrouw *et al*. (2009) [14]	8–9 yr SP	−0.93 ± 1.95	8–9 yr NSP	−1.11 ± 2.10	Only SP in the 16–18 age group had higher ICD-IMD than controls in the same age group.
	10–11 yr SP	−0.26 ± 1.91	10–11 yr NSP	−0.99 ± 2.44	
	12–13 yr SP	−0.36 ± 1.99	12–13 yr NSP	−0.75 ± 2.38	
	14–15 yr SP	0.80 ± 2.06	14–15 yr NSP	0.52 ± 2.91	
	16–18 yr SP	2.57 ± 2.11	16–18 yr NSP	1.90 ± 2.31	
Žlak *et al*. (2024) [25]	12 yr SP	0.00 ± 1.62	12 yr IHP	−0.64 ± 2.72	ICD-IMD increased with age. No statistically significant difference was found in comparisons between the same age groups.
	13 yr SP	1.23 ± 2.03	13 yr IHP	0.35 ± 1.67	
	14 yr SP	2.33 ± 2.25	14 yr IHP	2.68 ± 3.06	
	15 yr SP	2.71 ± 2.33	15 yr IHP	2.60 ± 1.09	
	16 yr SP	3.40 ± 1.99	16 yr IHP	3.10 ± 1.31	
De Cock *et al*. (2018) [18]	Boys	1.59 ± 2.20	Girls	1.23 ± 2.26	For boys, there are significant differences across the three age groups. For girls, only the other two age groups differ significantly from 12–13 years. There are significant differences between boys and girls at ages 12–13 and 16–18 years.
	12–13 yr boys	0.00 ± 1.93	12–13 yr girls	0.56 ± 2.07	
	14–16 yr boys	1.52 ± 1.82	14–16 yr girls	1.55 ± 2.24	
	16–18 yr boys	2.74 ± 1.91	16–18 yr girls	1.30 ± 2.30	
Yaniv *et al*. (2006) [24]	10–13 yr SP	0.86	10–13 yr TP	0.43	Only SP in the 14–16 age group had higher ICD-IMD than TP in the same age group.
	14–16 yr SP	3.64	14–16 yr TP	1.42	
Krajnc *et al*. (2015) [27]	SP	2.36 ± 1.62	No control group	-	ICD-IMD of SP is 2.36 cm.
Memmel *et al*. (2024) [21]	SP	−0.40 ± 3.30	No control group	-	ICD-IMD is significantly different among all age groups. The highest ICD-IMD level is in the 15–18 age group.
	7–11 yr SP	−2.00 ± 3.10	No control group	-	
	12–14 yr SP	−0.40 ± 3.10	No control group	-	
	15–18 yr SP	1.60 ± 2.40	No control group	-	
Schulz *et al*. (2025) [22]	U12 SP	0.70 ± 0.90	No control group	-	The highest ICD-IMD level is in the U17 age group. Genu varum may be associated with an increased risk of injury.
	U13 SP	1.00 ± 1.30	No control group	-	
	U14 SP	0.90 ± 1.20	No control group	-	
	U15 SP	1.50 ± 1.20	No control group	-	
	U16 SP	2.50 ± 1.90	No control group	-	
	U17 SP	3.20 ± 2.30	No control group	-	
Steffen *et al*. (2025) [23]	U12 SP	−0.56 ± 2.25	No control group	-	The highest ICD-IMD level is in the U17 age group.
	U13 SP	−0.47 ± 2.57	No control group	-	
	U14 SP	−0.32 ± 2.06	No control group	-	
	U15 SP	1.18 ± 2.35	No control group	-	
	U16 SP	1.40 ± 2.81	No control group	-	
	U17 SP	2.20 ± 2.56	No control group	-	

SP, soccer players; NSP, non-soccer players; IHP, ice hockey players; TP, tennis players; ICD-IMD, intercondylar-intermalleolar distance.

In studies, ICD-IMD values of athletes were reported between −1.91 and 5.65 cm. Differences in ICD-IMD measurements have been observed across younger age groups in the reviewed studies. While some studies reported negative ICD-IMD values in these populations [14, 15, 21, 23], others found positive values [6, 18, 22, 24, 25].

### Radiological measurement outcomes

Table 4 summarizes the measurements used in the studies, including hip–knee–ankle angle (HKA), mechanical lateral distal femoral angle (mLDFA), medial proximal tibial angle (MPTA), lateral proximal femoral angle (LPFA), mechanical lateral proximal femoral angle (mLPFA), medial proximal tibial physeal angle (MPTPhyA) and lateral distal femoral physeal angle (LDFPhyA). In these studies, mean HKA values generally varied in the varus direction. MPTA values were reported to be lower in athletes compared to control groups, which may be related to the development of genu varum [4, 5, 26]. On the other hand, no consistent trend was found between the studies in terms of LPFA values [4], with LPFA values were reported as higher in athletes in some studies and lower in others compared to the control group [26].

**Table 4:** Radiological measurement outcomes reported in the included studies.

Author (year)	Measured parameters	Group	Mean ± SD	Control	Mean ± SD	Main outcomes
Colyn *et al*. (2016) [4]	HKA	10–12 yr SP	−2.0 ± 2.4	10–12 yr OSP	−3.2 ± 2.2	Low MPTA contributes the most to varus alignment. The proximal tibial physis is likely to be one of the main mechanisms in the development of varus alignment.
		13–14 yr SP	−2.2 ± 2.4	13–14 yr OSP	−2.7 ± 2.5	
		15–17 yr SP	−1.8 ± 2.4	15–17 yr OSP	−3.1 ± 2.3	
	mLDFA	10–12 yr SP	88.2 ± 1.8	10–12 yr OSP	88.5 ± 1.3	
		13–14 yr SP	87.9 ± 1.8	13–14 yr OSP	88.6 ± 1.3	
		15–17 yr SP	87.8 ± 1.8	15–17 yr OSP	88.5 ± 1.1	
	MPTA	10–12 yr SP	85.8 ± 2.1	10–12 yr OSP	86.5 ± 3.3	
		13–14 yr SP	85.9 ± 2.3	13–14 yr OSP	86.7 ± 2.2	
		15–17 yr SP	85.6 ± 2.1	15–17 yr OSP	87.0 ± 1.1	
	LPFA	10–12 yr SP	88.1 ± 5.0	10–12 yr OSP	85.9 ± 6.1	
		13–14 yr SP	88.4 ± 5.2	13–14 yr OSP	86.3 ± 5.5	
		15–17 yr SP	88.5 ± 5.1	15–17 yr OSP	85.8 ± 5.6	
Işın *et al*. (2025) [26]	HKA	SP with genu varum	−4.2 ± 1.8	SP without genu varum	−1.6 ± 0.8	Factors contributing to genu varum formation differ in the dominant (LDTA) and non-dominant leg (MPTA). Proximal tibia is likely to be one of the main mechanisms in the development of varus alignment.
	mLDFA	SP with genu varum	88.2 ± 1.5	SP without genu varum	86.5 ± 1.6	
	MPTA	SP with genu varum	84.6 ± 3.8	SP without genu varum	85.5 ± 2.3	
	mLPFA	SP with genu varum	89.3 ± 2.8	SP without genu varum	91.6 ± 3.0	
Fernquest *et al*. (2023) [5]	HKA	11–12 yr male SP	0.9 ± 1.5	11–12 yr male controls	0.4 ± 1.8	A lower MPTPhyA value may be effective in the development of varus leg alignment. The proximal tibial physis is considered to play an important role in genu varum formation.
		13–14 yr male SP	0.9 ± 2.4	13–14 yr male controls	1.5 ± 1.4	
		15–16 yr male SP	−1.5 ± 2.7	15–16 yr male controls	−0.1 ± 1.5	
		17–18 yr male SP	−2.5 ± 1.4	17–18 yr male controls	−0.6 ± 1.3	
	MPTPhyA°	11–12 yr male SP	90.9 ± 2.1	11–12 yr male controls	92.9 ± 1.9	
		13–14 yr male SP	92.9 ± 2.7	13–14 yr male controls	92.7 ± 2.3	
		15–16 yr male SP	90.4 ± 2.6	15–16 yr male controls	93.1 ± 2.8	
		17–18 yr male SP	89.2 ± 3.4	17–18 yr male controls	92.3 ± 3.3	
	LDFPhyA	11–12 yr male SP	86.4 ± 2.8	11–12 yr male controls	86.0 ± 2.3	
		13–14 yr male SP	86.1 ± 3.0	13–14 yr male controls	85.1 ± 3.1	
		15–16 yr male SP	84.4 ± 3.9	15–16 yr male controls	83.2 ± 5.0	
		17–18 yr male SP	86.9 ± 2.4	17–18 yr male controls	83.9 ± 3.2	

HKA, hip–knee–ankle angle; mLDFA, mechanical lateral distal femoral angle; MPTA, medial proximal tibial angle; LPFA, lateral proximal femoral angle; mLPFA, mechanical lateral proximal femoral angle; MPTPhyA, medial proximal tibial physeal angle; LDFPhyA, lateral distal femoral physeal angle; SP, soccer players; OSP, other sport players.

## Discussion

This systematic review aimed to examine the relationship between genu varum and sport participation among youth athletes. Most of the included studies were either entirely focused on soccer or consisted of designs in which soccer was evaluated in combination with other sports. Accordingly, the findings were predominantly interpreted in terms of knee alignment in male soccer players. This is not only a consequence of the data distribution but also related to the fact that soccer is the most widely participated sport in younger age groups and genu varum is reported more frequently in this population. Nevertheless, current studies suggest that there are also slight trends towards varus alignment in non-soccer athletes, but this trend is not as pronounced as in male soccer players [6, 7, 14, 24].

The results of our research add to the growing body of literature that shows that genu varum occurs more frequently among young male athletes involved in high levels of physical activity compared to peers who do not participate in sports [4, 5, 19]. This is particularly true for young male soccer players, which is likely due to the specific loading patterns in soccer. This is concerning as varus malalignment predisposes athletes to abnormal biomechanical loading of the medial tibiofemoral joint, traumatic chondral and meniscal lesions, and premature medial joint osteoarthritis [8, 23, 25].

Biomechanical overloading of the proximal medial tibial physis has been proposed as a possible pathomechanism of genu varum in youth soccer players [5]. Soccer playing, particularly at higher competitive levels, induces an inordinate amount of load and torque in the lower extremity [14, 28]. In a growing skeleton, these forces are transferred to growth plates of the knee that are subsequently asymmetrically impacted [27]. This effect may be most pronounced if excessive loading occurs during the growth spurt, most notably during peak height velocity, or during the latter part of the growth spurt, just before physeal closure, when the bone is still actively growing, the cartilage is mechanically weakest, and activity-related loads are at their peak [4, 5, 29].

It is reasonable to expect that an emphasis on year-round training and competition as well as single-sport specialization helps to set the stage for PSI. The most common radiographic or magnetic resonance imaging finding is widening of the epiphyseal primary physis [30]. This finding is believed to reflect an alteration in metaphyseal perfusion and metaphyseal stress injury that occurs with repetitive loading, which interferes with the apoptosis of the hypertrophied chondrocytes [30, 31]. The hypertrophic zone continues to widen because of continued growth in the germinal and proliferative zones [30, 32].

When diagnosed and treated early, epiphyseal PSIs are usually reversible following the restoration of metaphyseal perfusion [30, 33, 34]. However, undiagnosed or sub-optimally treated epiphyseal PSIs predispose the growth plate complex to ongoing trauma and injury that can ultimately lead to injury of the physis proper and juxtaphyseal epiphysis [35]. Injuries to these latter components are typically irreversible and can manifest in asymmetric physeal growth, premature physeal closure, produce angular deformity, or even overgrowth depending upon the severity and/or duration of the insult [34]. In the young soccer player, the growth insult may be localized causing asymmetric growth pursuant to the mechanical overload of proximal medial tibial physis [36].

### Areas of agreement

Genu varum is the most common lower extremity malalignment among professional soccer players. The prevalence of genu varum in former professional soccer players varies between 55.6%–63% [37], with some studies reporting a prevalence of approximately 73% [28]. Similarly, 67.7% of young athletes have been reported to suffer from genu varum [19], indicating that lower limb malalignment is common both during youth and throughout the professional soccer career.

Previous studies revealed that measure of genu varum in athletes showed significant relationships with height, body weight, age, body mass index (BMI), and duration of participation in sports. The findings show that as height, body weight, age, BMI and duration of sports participation increase, the degree of genu varum also increases [6, 7, 38], perhaps reflecting a dose–response relationship. As height increases, the mechanical loads to which bones and joints are exposed during growth also increase. Especially during periods of rapid growth, the load-bearing axis of the knees may temporarily change. Increased body weight and BMI can increase the load on the knee joint, causing more pressure in the medial knee. Indeed, the medial compartment carries more load than the lateral compartment and the effect of weight changes is more pronounced in this area [39]. Furthermore, long-term sports participation including repetitive loads and high intensity exercises can lead to changes in the knee axis and contribute to an increase in the degree of genu varum [38].

More detailed analyses using radiological assessments suggest that decreased MPTA may be an important factor associated with increased varus alignment in soccer players [4, 26]. The MPTA is approximately 85° in both youth [26] and professional soccer players [40]. In contrast, soccer players with genu varum, defined by an ICD-IMD of 4.0 cm or greater, may exhibit MPTA values as low as 83.5° [12, 26]. These study results suggest that the proximal tibia may play an important role in lower-extremity malalignment among soccer players [4–6, 12, 26]. They also suggest that the morphologic and angular characteristics of the proximal tibia may influence knee joint load distribution and mechanical axis, leading to lower-extremity malalignment.

### Areas of controversy

In the studies reviewed, differences in ICD-IMD measurements were observed across younger age groups. Some studies reported negative ICD-IMD values in these age groups [14, 15, 21, 23]. On the other hand, positive ICD-IMD values were reported in other studies [6, 18, 22, 24, 25]. These findings may reflect the effects of different age groups studied, measurement methods, or sample characteristics on ICD-IMD values. They also reflect the absence of a consistent trend across studies regarding the direction of leg alignment at young ages, with some children showing varus alignment and others valgus alignment.

Generally, soccer players have been reported to show higher varus alignment compared to their non-athletic peers. The increase in varus is especially evident in adolescence [6, 14, 15]. In the study conducted by Işın and Melekoğlu [6], higher varus levels were reported in soccer players in all age groups between 10 and 18 years. On the other hand, some studies reported that this difference only became significant after a specific age [14, 15]. For example, Thijs *et al*. [15] reported that it became significant after 13 years of age, whereas Witvrouw *et al*. [14] reported that it became significant in the 16–18 age group. These differences may be related to variation in study designs, characteristics of participants, measurement methods, and duration of sports participation.

Some studies suggest that repetitive soccer activities, such as running, passing, changing direction, tackling, and kicking, may be affect knee alignment over time [6, 7, 14]. These movements may increase the mechanical load on the lower limbs during childhood and could be linked to the development of genu varum. However, most current evidence relies on ICD-IMD measurements, which do not fully explain alignment or reveal the underlying bone structure. Muscle strength imbalances, particularly between the hip adductor and abductor muscle groups, may also be related to genu varum. For example, Işın *et al*. [26] found that soccer players with genu varum often exhibit an imbalance between these muscle groups, but further research is needed to confirm this association.

In addition to the proposed role of MPTA in genu varum among soccer players, a recently published study suggested that different alignment characteristics may be associated with varus alignment in the dominant and non-dominant legs [26]. Specifically, LDTA in the dominant leg and MPTA in the non-dominant leg were identified as potential factors associated with varus alignment. These findings suggest that different alignment patterns may be present between the kicking and supporting legs [26]. However, the underlying mechanisms and long-term effects of sport-related loading on both legs remain unclear.

Between sport comparisons yield different results. Žlak *et al*. [25] found no difference in varus alignment between soccer and ice hockey players, whereas Yaniv *et al*. [24] reported greater varus alignment in soccer players after age 14 than in tennis players. Memmel *et al*. [20] found no significant difference in varus alignment between adolescent soccer players and referees. Evidently, the emergence and prevalence of varus alignment in athletes may vary not only by sport but also by age at the onset of the sport, duration and timing of exposure relative to peak height velocity (i.e. Pre-PHV, circa PHV, post-PHV), and mechanical loads during the adolescent growth period. Differences between sports become more noticeable after age 14. This suggests that repeated mechanical loading during skeletal growth might affect lower-extremity alignment while the growth plates are still open. Still, the exact reasons for these changes remain unclear, as there is little radiological evidence from sports other than soccer. As a result, it is not certain whether these differences are due to the specific ways each sport loads the body or to other factors, such as how long someone plays, the type of loading, or growth-related changes.

### Growing points

Early studies investigating varus deformity in youth athletes frequently used calipers or tape to determine ICD-IMD instead of radiologic measurements [6, 14, 15, 21, 22, 24]. Although these studies helped to explain the level of genu varum in athletes, it was not possible to determine the underlying mechanisms. In recent years, there has been an increasing number of radiological evaluations, allowing a more detailed analysis of the factors contributing to the genu varum development.

### Areas timely for developing research

Long-term follow-up and imaging studies are needed to better clarify the frequency and etiology of genu varum in young athletes. In this regard, future longitudinal studies should assess sports exposure together with measures of biological maturation rather than relying primarily on chronological age. The timing of exposure relative to peak height velocity, growth velocity, and skeletal maturation may be particularly relevant. The lack of longitudinal data examining the effects of sports loads on lower limb alignment during growth is a major limitation.

The relationship between genu varum and sports other than soccer has not been adequately investigated. Therefore, movement and loading characteristics specific to different sports should be evaluated. In addition, early detection of genu varum in young athletes is of high importance in terms of preventing knee osteoarthritis and overuse injuries that may occur later in life. In this regard, possible contributions of training volume and intensity, footwear selection, and sport specific technical requirements to the development of genu varum should be evaluated.

Since research has predominantly focused on male athletes [7, 19, 25], further research is needed to examine whether female soccer players are characterized by more frequent genu varum, especially given the difference in pelvic structural variations.

Although the number of radiological studies has increased in recent years [4, 5, 26], there is still a need for more radiological research, including athletes representing of a range of sports. Indeed, such research findings may provide more detailed information on the underlying mechanisms of varus deformities and contribute to the development of clinical intervention strategies. In addition, conventional radiologic imaging is usually performed with patients in a supine position. In this case, the effect of gravity on the knee cannot be considered [26] since knee alignment in the actual standing position is not accurately reflected. In future studies, evaluation of the deformity with weight-bearing radiologic measurements may prove more accurate.

Lastly, another area that should be studied in the future and can be addressed within the scope of longitudinal studies is the effect of genu varum development on stature and sitting height. Preliminary study on this topic suggests that there may be a link between playing soccer, notably during the growth period, and slowed bone growth in the left leg in a left-legged patient, and in the right leg in a right-legged patient [41].

### Limitations of the review

There are some limitations to this study. First, the studies reviewed were mostly observational and cross-sectional in design. Cross-sectional studies provide a snapshot of a population at a single point in time, allowing the prevalence of exposures and outcomes to be assessed. While this design is useful for identifying relationships, it does not allow for determining the sequence of events between exposure and outcome due to the lack of temporal data. This leads to uncertainty about the direction of causality. Most of the available evidence stemmed from studies involving soccer players (14 of the 16 included studies). Therefore, this should be considered when interpreting the findings. In addition, sample sizes between studies vary considerably and some studies include relatively small cohorts, which may limit the generalizability of results. A further limitation is that the methods used for angle measurements (image-based and radiologic evaluation) and the reference angles differ, which limits direct comparison of the findings. Finally, this systematic review is limited to studies published in English.

## Conclusion

The proximal tibia may be an important contributing factor in the development of genu varum in youth athletes. High levels of physical activity may contribute to the development of varus alignment by increasing skeletal adaptation and risk of injury involving the proximal tibia. Therefore, monitoring lower-limb alignment should be considered when clinically indicated in children participating in intensive sports during adolescence. Finally, longitudinal radiologic and epidemiological studies are needed to better elucidate the association between youth sport and genu varum and to determine causality. In this regard, it will be important to assess sports exposure together with measures of biological maturation rather than relying primarily on chronological age.

## Supplementary Material

Supplementary_material_ldag033

## Data Availability

No new data were generated or analyzed in support of this review.
