# Supplementary material for: Impact of sports participation on genu varum among youth: a systematic review
Source: Br Med Bull. 2026 Oct 5;160(1):ldag033. doi: 10.1093/bmb/ldag033 (PMC13662607; doi:10.1093/bmb/ldag033)
Supplement: Supplementary file 1 [file supplementary_material_ldag033.zip › Supplementary_Table_S1_ldag033.docx]

**Supplementary Table S1:** Search Strategy

| **Search number** | **Search strategy** | **Result Number** |
| --- | --- | --- |
| **Embase (OVID)** | | |
| 1 | genu varum.ti,ab. | 822 |
| 2 | bowleg*.ti,ab. | 145 |
| 3 | varus knee malalignment.ti,ab. | 28 |
| 4 | lower leg alignment.ti,ab. | 29 |
| 5 | leg deformit*.ti,ab. | 248 |
| 6 | lower limb deformit*.ti,ab. | 491 |
| 7 | lower limb malalignment.ti,ab. | 92 |
| 8 | 1 or 2 or 3 or 4 or 5 or 6 or 7 | 1,780 |
| 9 | youth.ti,ab. | 135,438 |
| 10 | young.ti,ab. | 901,256 |
| 11 | adolescent*.ti,ab. | 507,081 |
| 12 | sport*.ti,ab. | 160,234 |
| 13 | soccer.ti,ab. | 14,945 |
| 14 | football.ti,ab. | 17,993 |
| 15 | basketball.ti,ab. | 8,075 |
| 16 | volleyball.ti,ab. | 3,808 |
| 17 | athlete*.ti,ab. | 97,346 |
| 18 | 9 or 10 or 11 or 12 or 13 or 14 or 15 or 16 or 17 | 1,578,459 |
| 19 | 8 and 18 | **260** |
| **Web of Science** | | |
| 1 | (((((((((TS = "Genu varum") OR (TS = bowleg*)) OR (TS = "varus knee malalignment")) OR (TS = "lower leg alignment")) OR (TS = "lower leg malalignment")) OR (TS = "leg deformit*")) OR (TS = "lower limb deformit*")) OR (TS = "lower limb malalignment"))  AND  (((((((((TS = youth) OR (TS = young)) OR (TS = adolescent)) OR (TS = sport*)) OR (TS = soccer)) OR (TS = football)) OR (TS = basketball)) OR (TS = volleyball)) OR (TS = athlete*))) | **372** |
| **SCOPUS** | | |
| 1 | (TITLE-ABS-KEY("genu varum") OR TITLE-ABS-KEY("bowleg*") OR TITLE-ABS-KEY("varus knee malalignment") OR TITLE-ABS-KEY("lower leg alignment") OR TITLE-ABS-KEY("lower leg malalignment") OR TITLE-ABS-KEY("leg deformit*") OR TITLE-ABS-KEY("lower limb deformit*") OR TITLE-ABS-KEY("lower limb malalignment")  AND  TITLE-ABS-KEY(youth) OR TITLE-ABS-KEY(young) OR TITLE-ABS-KEY(adolescent) OR TITLE-ABS-KEY(sport*) OR TITLE-ABS-KEY(soccer) OR TITLE-ABS-KEY(football) OR TITLE-ABS-KEY(basketball) OR TITLE-ABS-KEY(volleyball) OR TITLE-ABS-KEY(athlete*)) | **659** |
| **PubMed** | | |
| 1 | ("genu varum"[Title/Abstract] OR "bowleg*"[Title/Abstract] OR "varus knee malalignment"[Title/Abstract] OR "lower leg alignment"[Title/Abstract] OR "leg deformit*"[Title/Abstract] OR "lower limb deformit*"[Title/Abstract] OR "lower limb malalignment"[Title/Abstract])  AND  ("youth"[Title/Abstract] OR "young"[Title/Abstract] OR "adolescent"[Title/Abstract] OR "sport*"[Title/Abstract] OR "soccer"[Title/Abstract] OR "football"[Title/Abstract] OR "basketball"[Title/Abstract] OR "volleyball"[Title/Abstract] OR "athlete*"[Title/Abstract]) | **159** |
| **CINAHL** | | |
| 1 | ((TI "genu varum" OR AB "genu varum") OR (TI bowleg* OR AB bowleg*) OR (TI "varus knee malalignment" OR AB "varus knee malalignment") OR (TI "lower leg alignment" OR AB "lower leg alignment") OR (TI "lower leg malalignment" OR AB "lower leg malalignment") OR (TI "leg deformit*" OR AB "leg deformit*") OR (TI "lower limb deformit*" OR AB "lower limb deformit*") OR (TI "lower limb malalignment" OR AB "lower limb malalignment"))  AND  ((TI youth OR AB youth) OR (TI young OR AB young) OR (TI adolescent OR AB adolescent) OR (TI sport* OR AB sport*) OR (TI soccer OR AB soccer) OR (TI football OR AB football) OR (TI basketball OR AB basketball) OR (TI volleyball OR AB volleyball) OR (TI athlete* OR AB athlete*)) | **69** |
