# Supplementary material for: Impact of sports participation on genu varum among youth: a systematic review
Source: Br Med Bull. 2026 Oct 5;160(1):ldag033. doi: 10.1093/bmb/ldag033 (PMC13662607; doi:10.1093/bmb/ldag033)
Supplement: Supplementary file 1 [file supplementary_material_ldag033.zip › Supplementary_Table_S2_ldag033.docx]

**Supplementary Table S2.** JBI methodological quality appraisal of the included studies

| **Author name** | **JBI Checklist Type** | **Score** | **%** |
| --- | --- | --- | --- |
| Asadi et al. (2015) [7] | Cross-sectional study | 7/8 | 87.5 |
| Colyn et al. (2016) [4] | Cross-sectional study | 7/8 | 87.5 |
| De Cock et al. (2018) [18] | Cross-sectional study | 7/8 | 87.5 |
| Rezende et al. (2011) [19] | Cross-sectional study | 6/8 | 75 |
| Fernquest et al., (2023) [5] | Cross-sectional study | 8/8 | 100 |
| Işın et al. (2025) [26] | Case‑control | 9/10 | 90 |
| Işın and Melekoğlu (2020) [6] | Cross-sectional study | 7/8 | 87.5 |
| Krajnc et al. (2015) [27] | Case-series | 8/10 | 80 |
| Memmel et al. (2023) [20] | Cross-sectional study | 8/8 | 100 |
| Memmel et al. (2024) [21] | Cross-sectional study | 8/8 | 100 |
| Schulz et al. (2025) [22] | Cross-sectional study | 7/8 | 87.5 |
| Steffen et al., (2025) [23] | Cross-sectional study | 8/8 | 100 |
| Thijs et al., (2012) [15] | Cross-sectional study | 7/8 | 87.5 |
| Witvrouw et al. (2009) [14] | Retrospective cohort study | 7/11 | 63.64 |
| Yaniv et al. (2006) [24] | Cross-sectional study | 7/8 | 87.5 |
| Žlak et al. (2024) [25] | Cross-sectional study | 8/8 | 100 |
